# Methicillin-Resistant *Staphylococcus aureus* (MRSA) in Dairy Products and Bulk-Tank Milk (BTM)

**DOI:** 10.3390/antibiotics13070588

**Published:** 2024-06-25

**Authors:** Camino González-Machado, Rosa Capita, Carlos Alonso-Calleja

**Affiliations:** 1 Department of Food Hygiene and Technology, Veterinary Faculty, University of León, E-24071 León, Spain; 2 Institute of Food Science and Technology, University of León, E-24071 León, Spain

**Keywords:** MRSA, review, milk, dairy products

## Abstract

In order to contribute to an assessment of the role of food in the risks of transmission of methicillin-resistant *Staphylococcus aureus* (MRSA), a review was undertaken of research on this microorganism in milk and dairy products published from January 2001 to February 2024. A total of 186 publications were selected, 125 for dairy products and 61 for bulk-tank milk (BTM). MRSA was detected in 68.8% of the research into dairy products and 73.8% of investigations relating to BTM, although in most studies the prevalence was less than 5%. Of the set of *S. aureus* strains isolated, approximately 30% corresponded to MRSA. The foods most extensively contaminated with this microorganism were raw milk and some types of soft cheese. Determination of the *mec*A gene on its own is known not to suffice for the detection of all MRSA strains. The great diversity of techniques used to study MRSA in milk and dairy products made it difficult to draw comparisons between studies. It would thus be advisable to develop a standardized protocol for the study of this microorganism in foods.

## 1. Introduction

Methicillin-resistant *Staphylococcus aureus* (MRSA) has become a major agent of severe nosocomial infections, with a high fatality rate [1]. MRSA is usually resistant to multiple drugs, and especially to most beta-lactam antibiotics, as a result of the synthesis of a modified penicillin-binding protein (PBP2a), encoded by the *mec*A gene included in the SCC*mec* or staphylococcal cassette chromosome *mec* [2]. The *mec*A gene has been detected in the majority of MRSA isolates from animals, humans, and the environment. Hence, although there are other genes (*mec*B, *mec*C, *mec*D) that are also responsible for methicillin resistance in strains of the *Staphylococcaceae* family, detection of the *mec*A gene by polymerase chain reaction (PCR) is the technique usually employed to identify MRSA. Among the different existing phenotypic methods for detecting MRSA, latex agglutination has the highest sensitivity and specificity, followed by cephoxitin disc diffusion [3]. The use of selective media and chromogenic agar media containing cefoxitin may be useful for the identification of *Staphylococcus aureus* and MRSA. It has been suggested that the cefoxitin disk diffusion method has higher sensitivity and specificity for the detection of MRSA than the Chrom agar MRSA method [4].

MRSA strains are classified on the basis of their epidemiological origin as (1) hospital-associated or HA-MRSA, (2) community-associated or CA-MRSA, and (3) livestock-associated or LA-MRSA [5]. The earliest infections by MRSA were nosocomial; however, in the 1990s, MRSA infections began to be reported in people without prior exposure to hospital settings, leading to the identification of the community-associated type. MRSA has also been detected in cattle, with the first isolations made in Belgium in 1972 from bovine mastitis samples [6]. LA-MRSA has become a considerable potential menace to public health owing to its zoonotic capacities since it can be transmitted from animals to humans [2].

While the direct transmission of MRSA has been well studied, its role in food remains poorly understood. The classic food-borne staphylococcal disease is food poisoning resulting from the contamination of foodstuffs with enterotoxins [7]. Staff manipulating food who carry enterotoxin-producing *S. aureus*, which may be present in the nasal passages or on the skin, are seen as the main source of such contamination [8]. Staphylococcal food poisoning is mostly associated with improper handling of processed foodstuffs, followed by storage under conditions that allow the growth of *S. aureus* and the production of enterotoxins [9]. In contrast, food poisoning caused by strains of MRSA is very rare. The first outbreak of gastrointestinal illness caused by toxins produced by MRSA originated in the United States when an infected food handler caused the contamination of coleslaw [10]. From the studies cited above, it is clear that MRSA is present in food and can be a cause of food poisoning, and this fact poses a potential risk to human health. However, methicillin resistance is not a relevant factor for the production of enterotoxins and, since food poisoning is not a disease that is treated with antibiotics, MRSA should not pose, in this context, any greater risk than methicillin-susceptible *S. aureus*, that is, MSSA [11].

Another factor to be taken into account is the possibility of infection with MRSA during food processing or consumption. When food is cooked properly, the potential risk of infection is almost certainly minimal. However, the under-cooking of foodstuffs or their cross-contamination through contact with raw products or contaminated surfaces does pose a potential hazard to the consumer [9]. The risk depends largely on the hygiene measures adopted, the concentration of MRSA present, and the ability of the strain itself to colonize the host. In this respect, it should be noted that, in some studies, a low level of *S. aureus* was observed in the food samples analyzed [11]. Consequently, the transmission of MRSA to humans through the food chain is considered a minor route, and techniques that can detect very low levels of contamination are usually necessary to determine the microorganism [8].

Milk and dairy products are often contaminated with antibiotic-resistant bacteria, including *S. aureus*, which has become a critically important global public health problem [12]. In dairy animals, *S. aureus* is one of the most common causative agents of mastitis, and infected animals often excrete this bacterium in their milk. In recent years, interest in the consumption of minimally processed foods has increased, which is why the consumption of raw milk shows a growing trend. However, due to the possible presence of *S. aureus* in raw milk, this bacterium can be transmitted to humans through the dairy food chain and pose a risk to consumers [13]. The prevalence of *S. aureus* is higher in raw milk than in dairy products and pasteurized milk [12]. The hygiene and safety of raw milk can be assured by improving the health of dairy animals and hygienic practices during milking. Transportation and storage in accordance with regulatory requirements can reduce contamination of raw milk with *S. aureus* and staphylococcal enterotoxins [12]. Bulk tank milk has been identified as a source of MRSA, demonstrating the potential food safety risk of contaminated milk and dairy products entering the human food chain [14].

The objective of this work was thus to compile an overview of the literature available on the prevalence and types of MRSA in milk and dairy products, as well as to describe the methods used in each case. The intention was to elucidate what measures would allow better detection, identification and typing of strains.

## 2. Results

### 2.1. Dairy Products

**Figure 1:**
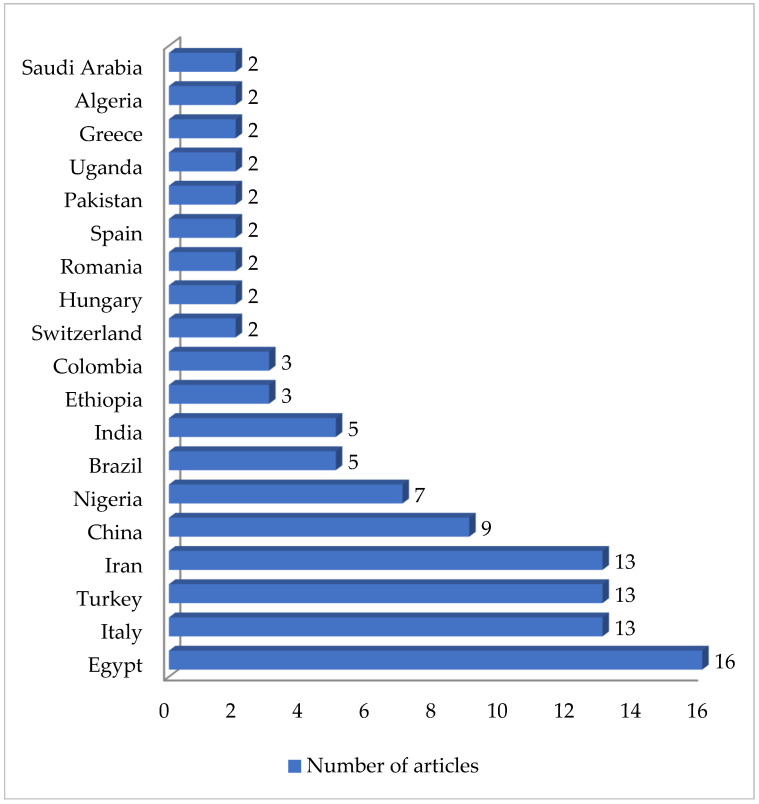
Research on MRSA in dairy products grouped by location.

**Figure 2:**
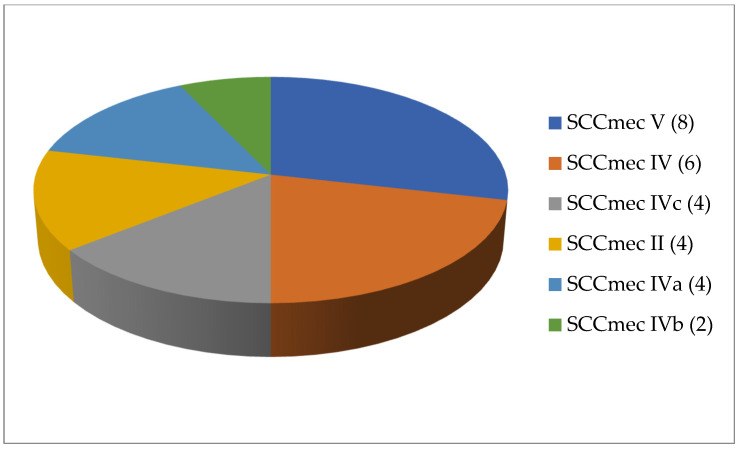
Most detected staphylococcal cassette chromosome *mec* (SCC*mec*) types in the reviewed research on MRSA in dairy products (number of articles is indicated in parentheses).

**Figure 3:**
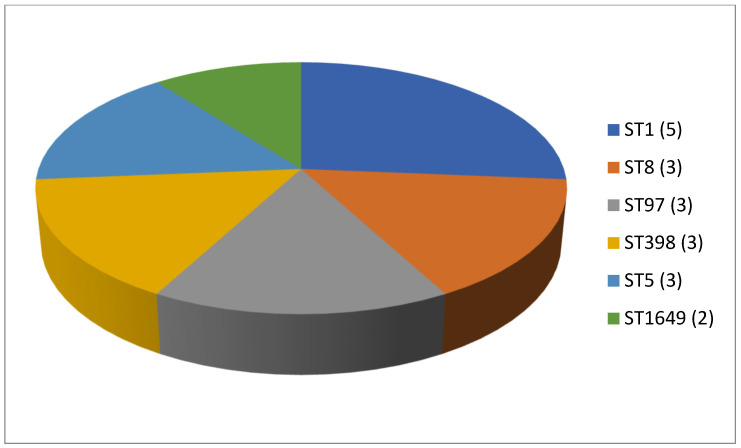
Most detected sequence types (ST) in the reviewed research on MRSA in dairy products (number of articles is indicated in parentheses).

A total of 125 articles relating to dairy products were reviewed (Table 1). No prior enrichment was performed in 55.2% of this work (69 cases out of 125). Few articles (4.8%, only 6 out of 125) described the application of double enrichment to the samples, and not one had triple enrichment. In most of the pieces of work using liquid media, 14 in all, tryptone soy broth (TSB) was used as a culture medium, and in 3 of these, the broth was supplemented with 7.5% salt (NaCl). In second place came Mueller Hinton broth (MHB) supplemented with 6.5% NaCl, which was used in 5 instances. With regard to solid media, only a few articles (4.8%; 6 out of 125) mentioned the use of a chromogenic medium selective for MRSA, and only 5 articles spoke of the employment of oxacillin resistance screening agar base (ORSAB) supplemented with oxacillin at 2 milligrams per liter (2 mg/L). Regarding the method for confirming MRSA, four main techniques were used. In a clear majority of the articles (67.2%, 84 out of 125), amplification of the *mec*A gene was achieved by using PCR. A total of 22 articles recorded amplification of the *mec*A and *mec*C genes by PCR, whilst in 10 articles disc diffusion tests for susceptibility to cephoxitin (30 μg) and oxacillin (1 μg) were utilized. Finally, in eight pieces of work MRSA latex agglutination testing, checking for penicillin-binding protein 2a, was employed. MRSA was found in 68.8% (86 out of 125) of the investigations, although in most of the work consulted the microorganism was detected in less than 5% of the samples. The percentage with multiple resistance was around 30% of the total *S. aureus* strains isolated. The most frequently contaminated foods were raw milk and some types of soft cheese. Regarding the location of the research works (Figure 1), the 4 most frequent countries were Egypt (16 articles), Italy, Turkey, and Iran (13 articles each). The most detected SCC*mec* and ST types in the different pieces of research were SCC*mec* V (8 articles) and IV (6); and ST1 (5) (Figure 2 and Figure 3).

### 2.2. Bulk-Tank Milk

**Figure 4:**
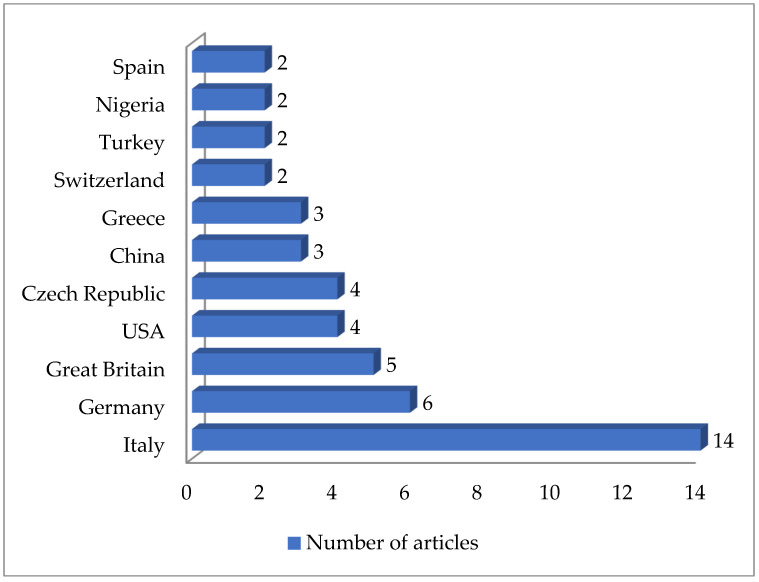
Research on MRSA in milk in bulk tankers grouped by location.

**Figure 5:**
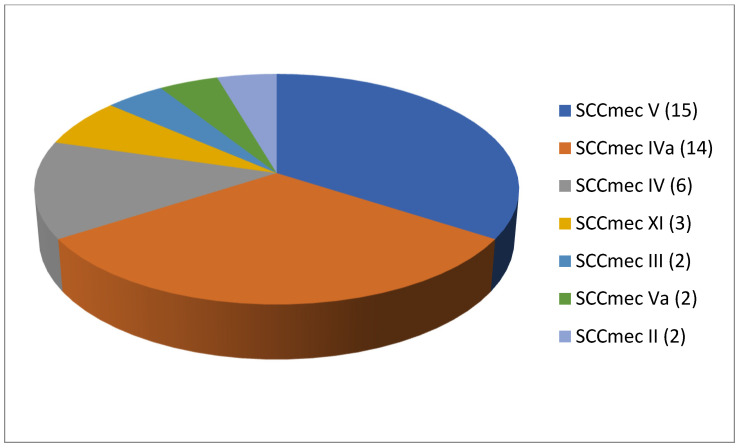
Most detected staphylococcal cassette chromosome *mec* (SCC*mec*) types in the reviewed research on MRSA in milk in bulk tankers (number of articles is indicated in parentheses).

**Figure 6:**
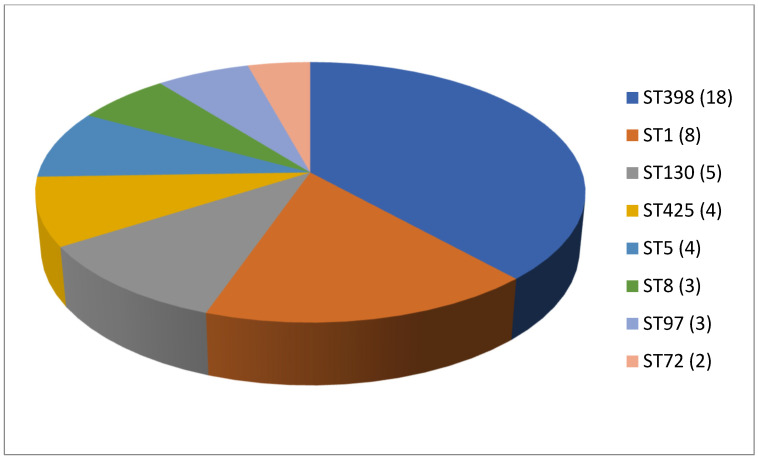
Most detected sequence types (ST) in the reviewed research on MRSA in milk in bulk tankers (number of articles is indicated in parentheses).

As shown in Table 2, 61 articles relating to milk in bulk tankers were reviewed. In an appreciable number of the pieces of research being reported (45.9%; 28 out of 61), no prior enrichment was performed for the isolation of MRSA. A quarter of the articles (24.6%, 15 out of 61) described double enrichment of the samples being carried out, but none mentioned triple enrichment. Regarding liquid media, MHB supplemented with 6.5% NaCl was used in 24 instances. The second commonest culture medium was TSB, mentioned in eighteen articles, with a majority of these, ten articles in total, recording supplementation with 75 mg/L of aztreonam and 3.5 mg/L of cephoxitin. With regard to solid media, some investigations (11.5%; 7 out of 61) used a selective chromogenic medium for MRSA, but only 2 referred to the use of agar-base for screening for resistance to oxacillin (ORSAB) supplemented with oxacillin (2 mg/L). As for the method of confirming MRSA, four main approaches were used. A majority of articles (59.0%, 36 out of 61) described amplification of the *mec*A gene by means of PCR. In 23 cases, there was amplification of the *mec*A and *mec*C genes through the use of PCR; in four, a disc diffusion test for susceptibility to cephoxitin (30 μg) and oxacillin (1 μg) was employed while in three instances the MRSA latex agglutination test, checking for penicillin-binding protein 2a, was used. MRSA strains were detected in 45 of the pieces of research, which was 73.8% of the total, although their prevalence was less than 5% in most of the reports consulted. Regarding the origin of the publications (Figure 4), it was observed that the four most frequent countries were Italy (14 articles), Germany (6 articles), and Great Britain (5 articles). The most frequently detected SCC*mec* and ST types are shown in Figure 5 and Figure 6. These were SCC*mec* V (15 articles), IVa (14), and IV (6); and ST398 (18), ST1 (8), and ST130 (5).

**Table 1:** Prevalence of MRSA in dairy products (in the absence of clarification, the prevalence is considered based on the detection of *mec*A gene).

Samples	Period	Place	Detection Method	Prevalence	Identification	Reference
350 staphylococcal isolates from food	2001	Hungary	-1.1. -2.12.1. -3.4.-3.7.-3.11.-3.2.	1% resistant to oxacillin from milk industry (*mec*A gene negative)	N/R	[15]
915 staphylococcal strains were isolated from foods examined at the National Food Investigation Institute	2001–2002	Hungary	-1.1. -2.12.1. -3.4.-3.7.-3.11.-3.2.	2 resistant to oxacillin: sausage and fresh milk (*mec*A gene negative)	N/R	[16]
216 isolates of *S. aureus* from raw milk samples	July 2001–February 2002	Rift Valley (Kenya)	-1.1. -3.4.	17/216 (7.8%) resistance to methicillin	N/R	[17]
641 milk and dairy products from retail outlets	January 2003–December 2005	Italy	-Homogenized: 1.2. -1.1. -2.1.2. -3.2.-3.4.	6/641 (0.9%): 4 from bovine milk and 2 from cheese (pecorino and mozzarella)	-3 strains belonged to the non-host-specific biovar -3 strains belonged to the ovine biovar	[18]
24 raw milk samples and 24 Minas Frescal cheese samples from a dairy processing plant	February 2004–March 2005	Goiás State (Brazil)	-1.1. -2.1. -3.4.	1 isolate resistant to oxacillin from Minas Frescal cheese	N/R	[19]
122 *S. aureus* strains came from different raw milk products (milk, curd, cheeses, butter, and whey): 81 isolates originated from cow, 22 from goat, 17 from sheep, and 2 from buffalo	N/R	Italy	-1.1. -3.1.	None	N/R	[20]
148 presumptive *S. aureus* isolates from: 9 cheeses, 18 bovine mastitis (from raw milk samples) and 20 raw cow’s milk	2006–2008	Portugal	-1.1. -2.1.2. -3.2.-3.4.	-38% of the isolates were resistant to oxacillin -1/148 (0.7%) showed the presence of *mec*A gene (from bovine mastitis in raw milk samples)	N/R	[21]
200 samples of raw milk cheese	March–September 2009	Switzerland	-First: 1.4.3. -Second: 1.11.1. -2.14. -3.2.-3.4.	None	N/R	[22]
36 *S. aureus* strains isolated from raw sheep’s milk cheese from dairies	N/R	Sardinia (Italy)	-1.1. -3.2.-3.4.	None	N/R	[23]
127 white-brined Urfa cheese samples from supermarkets and retail outlets	January–March 2008	Sanliurfa (Turkey)	-1.1. -2.1.1. -3.2.-3.4.	3/40 (7.5%) of *S. aureus* isolates	N/R	[24]
200 samples of raw milk, pasteurized milk, ice cream, traditional white pickled cheese from different supermarkets and retailer shops	March 2010–September 2011	Sarab (Iran)	-1.9. -2.1.3. -3.2.	4/200 (2%): 2 pasteurized milk and 2 traditional cheese	N/R	[25]
200 unpackaged cheese samples: 100 white cheese and 100 Tulum cheese from retail markets	April 2008–January 2009	Ankara (Turkey)	-1.1. -2.1.1. -3.2.-3.4.	2/200 (1%): Tulum cheese samples	N/R	[26]
300 samples from sellers: 100 raw milk, 100 pasteurized milk and 100 ice cream	During the summer in 2010	Tabriz (Iran)	-1.9. -2.1.3. -3.2.	-20/300 (6.7%): 14 raw milk and 6 ice cream samples -20/69 (29%) of *S. aureus* isolates	N/R	[27]
149 strains isolated from samples of food such as milk and milk derivatives	2004–2010	Colombia	-1.1. -2.1. -3.4.-3.2.	5/149 (3.4%): 1 milk cream	N/R	[28]
72 cheese samples (36 samples from dairy counters or refrigerated displays in grocery stores or farmer’s markets, and 36 samples from manufacturers via internet): 8 cream cheeses, 5 soft cheeses, 8 semi-hard cheeses, 1 hard and 1 processed cheese, produced from pasteurized milk, and 9 soft cheeses, 28 semi-hard- and 12 hard cheeses, made from raw milk	N/R	Hanover (Germany)	-1.4.1. -2.14. -3.2.	None	N/R	[29]
404 samples of Burrata Cheese from 12 different dairies	April–July 2009 and February–June 2010	Puglia (Italy)	-3.2.-3.4.	None	N/R	[30]
100 (56 fruity, 32 vanilla, and 12 chocolate) ice cream samples	June–September 2010	Samsun (Turkey)	-1.1. -2.1.1. -3.2.	-1/100 (1%): fruity ice cream -1/35 (2.8%) of *S. aureus* isolates	N/R	[31]
205 samples: 40 raw milk, 40 dairy products, and 20 ice cream from markets, grand large hotel and farms	April–November 2011	Anhui (China)	-1.1. -3.4.	None	N/R	[32]
35 raw milk, 30 Kariesh cheese, and 30 ice cream from local markets and villages	December 2011–February 2012	Dakahlia (Egypt)	-1.1. -3.4.-3.2.	5/95 (5.3%): 3 raw milk (8.6%), 1 Kariesh cheese (3.3%) and 1 ice cream (3.3%)	N/R	[33]
30 samples of cow raw milk and white raw soft cheese from markets	December 2012–February 2013	Baghdad	-Two methods: ·1.1. ·1.3.11. -2.1.4. -3.4.-3.9.	10/30 (33.3%): 4 raw milk (13.4%) and 6 white raw soft cheese with its whey (20%)	N/R	[34]
100 samples of Erzincan tulum cheese from markets	N/R	Erzincan (Turkey)	-Homogenized: 1.7. -1.1. -2.1. -3.2.-3.4.	10/61 (16.39%) of *S. aureus* isolates	N/R	[35]
347 samples of traditional and commercial dairy products from retail stores: 90 cheese, 85 ice-cream, 57 butter, 36 cream, 30 yoghurt, 24 kashk and 25 Iranian dough	September 2010–July 2011	Isfahan, Chaharmahal va Bakhtyari and Khuzestan (Iran)	-Homogenized: 1.10. -1.1. -2.1. -3.4.	Of 20 *S. aureus* isolates: 5 (25.0%) resistant to oxacillin and 0 to methicillin	N/R	[36]
160 milk and milk product samples (30 pedha and 30 curd) from milk collection center of Co-operative milk dairies, cattle farms, individual household, milk vendors, and sweet shops	February–October 2011	Anand, Gujarat (India)	-1.7. -2.1. -3.4.	None	N/R	[37]
372 milk samples: 300 raw milk, 17 each of pasteurized milk and yogurt, and 20 ‘kindirmo’ (locally fermented milk)	N/R	Kaduna and Zaria (Nigeria)	-1.1. -2.1. -3.4.-3.2.	-18/47 of coagulase positive *S. aureus* were resistant to methicillin (4.8%): 14 in raw milk and 1 each in pasteurized milk and ‘kindirmo’ -4/18 of the MRSA isolates (*mec*A gene positive): 3 raw milk and 1 ‘kindirmo’	N/R	[38]
340 raw milk samples from dairy farms and shops: 120 cow, 140 buffalo, 40 sheep and 40 goat	N/R	Sohag Governorate (Egypt)	-1.1. -2.4.-2.1. -3.4.	Resistance to Oxacillin (MRSA): 43.1% for cow, 40% buffalo, 8.7% sheep and 6.7% goat milk	N/R	[39]
100 Iranian white and feta cheese samples collected from different suppliers	N/R	Mashhad (Iran)	-1.1. -2.1.1. -3.4.-3.2.	8/23 (34.8%) of *S. aureus* isolates: 2 Iranian Feta cheese and 6 Traditional white cheese	N/R	[40]
623 isolates of CPS were isolated from 78 bovine raw milk cheeses by different food control Laboratories originated from: 48 cheese curd and 30 finished cheese	N/R	Switzerland	-1.1. -2.1.2.-2.3. -3.2.	1/623 (0.2%)	*spa* type t127 (human origin)	[41]
100 traditional cheeses samples	January–December 2012	Azerbaijan (Iran)	-1.1. -3.2.	23/110 (21%) of *S. aureus* isolates	N/R	[42]
94 *S. aureus* isolates: 87 cow’s milk with mastitis, 6 raw cheese, and 1 milking machine swab from 12 dairy farms	N/R	Pernambuco (Brazil)	-1.5. -2.4. -3.4.	None	N/R	[43]
150 *S. aureus* strains were collected from curd cheese manufactured at artisan level from raw sheep milk	N/R	N/R	-1.1. -2.1.2. -3.3.-3.4.	None	N/R	[44]
295 raw milk samples from supermarkets and farmers markets	July 2008–December 2012	Shaanxi (China)	-1.3.9. -2.1.1. -3.4.-3.2.	4/295 (1.4%)	- ST9/SCC*mec* IVb/t899 (4) - ST9/SCC*mec* II/t899 (1)	[45]
565 milk and dairy products samples were collected from bovine, ovine, caprine and bubaline farms: 428 raw milk, 9 thermised milk, 8 curd, 7 “Ricotta” cheese, 8 yoghurt and 105 cheese	2011–2013	Central Italy	-1.1. -3.2.-3.4.	2/227 (0.9%) of *S. aureus* isolates: 2 different “pasta filata” cheese samples	ST1 (CC1, human CA-MRSA)/*spa* type t127/SCC*mec* IVa	[46]
120 samples from retail markets, street vendors and free range (reared) sheep and goat flocks: 15 raw buffalos’ milk, 15 sheep milk, 15 goat milk, 15 yogurt, 30 Kareish cheese and 30 ice cream	N/R	Ismailia (Egypt)	-1.3. -2.2. -3.2.-3.4.	30/120 (25%): 5 goat milk (5/15, 33.3%), 3 sheep milk (3/15, 20%), 2 buffalo milk (2/15, 13.3%), 6 yoghurt (6/15, 40%), 4 ice cream (4/30, 13.3%) and 10 Kareish cheese (10/30, 33.3%)	N/R	[47]
68 CPS isolates obtained from 100 samples of artisan double cream cheese	N/R	Pamplona (Colombia)	-1.1. -2.1. -3.2.	-9/100 (9%) -12/66 (18.2%) of *S. aureus* isolates	N/R	[48]
2650 samples: 1035 raw cow milk, 895 raw sheep milk, 450 traditional cheese and 270 kashk (prepared by prolonged boiling yogurt) from retail stores	January 2006–December 2013	Mazandaran (Iran)	-Homogenized: 1.7. -1.1. -2.1.1. -3.4.-3.2.	53/2650 (2%): 21 cow milk, 11 sheep milk, 15 traditional cheese and 6 kashk	N/R	[49]
1069 bovine raw milk samples, 910 goat milk powder processing samples and 245 powdered infant formula	July 2008–March 2014	China	-First: 1.2. -Second: 1.3.4. -2.1.1. -3.4.-3.2.	None	N/R	[50]
1050 food samples: 671 dairy products (66 cream, 45 traditional yoghurt, 271 raw milk, 47 traditional cream, 170 cheese, and 72 butter)	September 2013–June 2014	Hamadan (Iran)	-1.12.1. -2.1.1. -3.4.-3.5.-3.2.	5/98 (5.1%) of *S. aureus* isolates: 3 raw milk and 2 cheese	N/R	[51]
200 food samples sold in a black market at an EU border: milk and dairy products (36%)	July 2012–February 2013	Galati (Romania)	-1.1. -3.4.-3.3.	None	N/R	[52]
383 raw milk samples from 101 vending machines	April–October 2012	Milan and Monza-Brianza (Italy)	-1.1. -2.1.2. -3.4.	7/383 (1.8%): 1.9% (2/101) from vending machines	- SCC*mec* IV (2 vending machines)	[53]
75 dairy products confiscated from passengers on flights from non-EU countries: 74 cheeses and 1 butter	April 2012–June 2013	International Bilbao Airport (Spain)	-1.1. -3.4.-3.3.	1/75 (1.3%): 1 cheese sample	SCC*mec* IVc/ST1649 (CC6) (CA-MRSA)	[54]
121 samples from different food markets: dairy products (1 pasteurized milk and 5 dry cakes)	August–October 2010	Wuhou District of Chengdu city, Sichuan Province (China)	-1.5. -2.1.1. -3.2.-3.4.	None	N/R	[55]
476 cow’s cheese and 453 raw cow’s milk for vending machine collected during official monitoring activities	2008	Italy	-1.1. -3.5.-3.4.	None	N/R	[56]
200 samples of raw milk and dairy products (Damietta cheese, Kareish cheese, ice cream, and yogurt) from retail outlets, different shops and supermarkets	June–November 2012	Mansoura (Egypt)	-1.1. -2.1.1. -3.2.-3.4.	106/200 (53%): raw milk (30/40, 75%), Damietta cheese (26/40, 65%), Kareish cheese (16/40, 40%), ice cream (20/40, 50%), and yogurt samples (14/40, 35%)	N/R	[57]
173 milk samples from apparently healthy and mastitic cows	December 2012– December 2013	Sharkia (Egypt)	-1.1. -3.4.-3.2.	30/173 (17.3%)	N/R	[58]
51 raw milk and 5 local cheese from retail markets	July 2012–March 2013	Kırşehir (Turkey)	-First: 1.2. -Second: 1.12. -2.1.1. -3.2.	None	N/R	[59]
90 samples of typical sheep and goat cheeses (soft and semi-hard canestrato, ricotta, and cacioricotta) from dairies	October 2013–April 2014	Southern Italy	-1.1. -2.1.2.-2.2. -3.2.	1/37 (2.7%) of *S. aureus* isolates: ricotta cheese (ovicaprine)	N/R	[60]
200 samples of raw and RTE Food illegally sold from a market at the Eastern EU Border: 73 dairy products (raw milk; cheeses made of cow, sheep, or goat raw or pasteurized milk, cream, and butter), from the Republic of Moldova, Ukraine, and Bulgaria	July 2012– February 2013	Galati (Romania)	-1.1. -2.1.2. -3.10.	0.03% Ewe cheese and Goat cheese	*spa* type t524	[61]
8 MRSA isolates, obtained from samples of fresh cheese (Doble Crema) elaborated from raw cow milk in small dairies collected at the retail level	April 2012–April 2013	Colombia	-1.1. -2.1.1. -3.3.-3.4.	Isolates from 8 cheese samples carried the *mec*A gene	ST8/SCC*mec* IV/t024 (related to the USA300, CA-MRSA)	[62]
56 CPS isolates recovered from samples of raw milk	N/R	Turkey	-Homogenized: 1.7. -1.1. -2.1. -3.5.-3.2.	2/56 (3.6%)	N/R	[63]
280 samples from markets: 140 yoghurt and 140 Nono	December–April	Kaduna (Nigeria)	-1.3.12. -2.1.1. -3.4.-3.9.-3.2.	-9/280 (3.2%) were identified using the Kirby-Baeur disk diffusion: 8 in nono (5.7%) and 1 yoghurt (0.7%) -6 (66.7%) were positive for the penicillin—binding protein and 7 (76.5%) were positive for betalactamase production - None of the 9 *S. aureus* was found to be positive for *mec*A	N/R	[64]
280 samples of yoghurt and nono (traditionally fermented milk) from major markets	December–April	Zaria (Nigeria)	-1.3.12. -2.1.1. -3.4.-3.9.-3.2.	-8/280 (2.9%) were identified using the Kirby–Baeur disk diffusion: 4 nono and 4 yoghurt -5/8 (62.5%) out of the 8 MRSA were positive for the penicillin—binding protein and 6 (75%) were positive for betalactamase production -None of the 8 MRSA harbored the *mec*A gene	N/R	[65]
910 samples: 62 raw goat milk samples from a milking station and 848 samples from seven different sampling sites in four goat milk powder processing plants (including tank milk, pre-spray drying areas, spray drying areas, powder-packaging room, ground and wall, workers, and final products)	September 2012–March 2013	Shaanxi (China)	-First: 1.2. -Second: 1.3.4. -2.1.1. -3.2.-3.4.	1/95 (1.1%) of *S. aureus* isolates: from the spray drying area (fluidized bed)	N/R	[66]
900 bovine milk samples from private and public farms: 450 cattle and 450 buffalo)	N/R	Faisalabad (Pakistan)	-1.1. -2.3. -3.7.-3.2.-3.4.	306/900 (34%): 135/450 (30%) cattle and 171/450 (38%) buffalo	N/R	[67]
148 milk samples, 117 ghee samples (butter from milk) and 91 sour milk samples (fermented milk) from 196 homesteads	July–August 2013	Sanga and Kanyaryeru (Uganda)	-Transportation: 1.5. -1.1. -2.4.-2.3. -3.3.-3.4.-3.11.	23/41 (56.1%) of the *S. aureus* isolates: milk (15/30, 50%) and sour milk (8/11, 72.7%) (none carried the *mec*C)	-SCC*mec* V (21/23, 91.3%): t2112 (3), t127 (2, ST1), t14299 (1), t645 (1, ST121), t1398 (2), t3992 (1, ST97), t3772 (1) and unknown (10) -SCC*mec* IV: unknown (1) -SCC*mec* IVc: t7753 (1)	[68]
3760 samples of milk and dairy products from several dairies	2008–2014	Apulia (Italy)	-1.1. -2.2. -3.2.	40/484 (8.3%) of *S. aureus* isolates: 2 mozzarella cheese, 1 stracciatella cheese, 20 cheese, 1 scamorza cheese and 16 milk	- ST152/t355/SCC*mec* V (27/40, 67.5%) (CA-MRSA) (stracciatella cheese, cheese and milk) -ST398/t899/SCC*mec* V (7/40, 20%) (LA-MRSA) (cheese) -ST398/t899/SCC*mec* NT (1/40, 2.5%) (LA-MRSA) (milk) - ST398/t108/SCC*mec* V (2/40, 5%) (LA-MRSA) (mozzarella cheese) -ST1/t127/SCC*mec* IVa (2/40, 5%) (human origin) (milk) -ST5/t688/SCC*mec* V (1/40, 2.5%) (HA- and CA-MRSA) (scamorza cheese)	[69]
71 traditional raw milk cheeses from local markets	N/R	Belgrade (Serbia)	-1.1. -2.1. -3.4.-3.3.	None	N/R	[70]
50 traditional cheeses (25 Carra and 25 Surk)	August 2014–May 2015	Hatay (Turkey)	-Homogenized: 1.7. -1.1. -2.1.1. -3.4.-3.2.	None	N/R	[71]
64 yoghurt samples from local bazaars and markets: 55 were unpackaged and 9 were packaged	March 2016	Şanlıurfa-Urfa (Turkey)	-1.1. -2.1.1. -3.2.	2/64 (3.1%): unpackaged samples	N/R	[72]
421 samples at different stages of processing from five dairies: 46 raw material, 161 food-contact surfaces, 96 non-food-contact surfaces, and 118 products	December 2013–July 2014	São Paulo, Minas Gerais and Goiás (Brazil)	-1.1. -2.1.1. -3.4.	None	N/R	[73]
175 samples: 125 dairy products samples (15 yoghurt, 40 white cheese, 10 kashar cheese, 15 tulum cheese, 12 mihalic cheese, 13 curd cheese, 10 sepet cheese, and 10 butter) from farms and retail markets	N/R	Balikesir (Turkey)	-1.4.3. -2.2. -3.9.-3.2.-3.4.	- 3/175 (1.7%) by Slidex MRSA latex agglutination test: 1 sample from tulum cheese -The *mec*A gene was detected in only one out of 17 *S. aureus* isolates (5.88%)	N/R	[74]
200 samples of dairy products from supermarkets: “requeijão”, “requeijão light”, “especialidade láctea type requeijão” and “especialidade láctea type requeijão light” (50 of each)	2015	Jaboticabal, São Paulo (Brazil)	-1.1. -2.1. -3.2.-3.4.	None	N/R	[75]
71 samples of raw milk, 212 samples of sour cream, 65 samples of home-made cottage cheese, and 57 washouts from the hands of the sellers of these products from food markets	N/R	Ukraine	-1.1. -2.1.-2.11. -3.2.	39/247 (15.8%) of *S. aureus* isolates	N/R	[76]
72 milk samples from mothers who had delivered in the hospital.	16 February 2014–24 April 2016	France	-1.1. -2.12.1. -3.2.-3.4.	Among the 62 *S. aureus* strains ten (16.2%) were phenotypically methicillin-resistant *S. aureus* (MRSA)	N/R	[77]
51 *S. aureus* strains were collected from 51 samples of 6 dairy sheep farms, including two farms with artisan dairy facilities, with a history of MRSA in BTM: 2 raw cheese samples	2013–2015	Latium (Italy)	-1.1. -2.1.2. -3.3.	27/51 (52.9%): 2 raw cheese	t127 (2)	[78]
69 bovine milk, whey and cheese samples from farm dairies	During a five-month winter period	Mid-Norway	-Homogenized: 1.7. -1.1. -2.1.1. -3.2.-3.4.	None	N/R	[79]
868 food samples of animal origin: 447 milk and dairy products confiscated from passengers on flights from 45 non-European Union (EU) countries by the Border Authorities, as well as 10 food products illegally introduced and sold in an open market closed to an EU border (the southeast part of Romania, on the border with Republic of Moldavia)	August 2012–July 2015	Bilbao International Airport (Spain) and Vienna International Airport (Austria)	-1.1. -2.1. -3.4.-3.3.	26/868 (3%): 21 milk and dairy products (cow, sheep or goat milk and cheese, either fresh, brined or with spices) (none harbored the *mec*C gene)	-The predominant sequence type was ST5 (30.8%) (HA and CA-MRSA), followed by ST8 (15.4%), ST1649 (15.4%) (both CA-MRSA), ST1, ST7, ST22, ST72, ST97, and ST398 (LA-MRSA) - More than 75% of the isolates were SCC*mec* type IV: 48.9% were IVc and IVe, 22.4% IVa, and 4.1% IVh, and 24.5% were SCC*mec* type V - Two isolates were not typeable by SmaI-PFGE suggesting they may be ST398 (livestock clone)	[80]
360 traditional cheese samples	N/R	Mazandaran (Iran)	-3.2.-3.4.	199/224 (88.8%) of *S. aureus* isolates	N/R	[81]
360 samples of pastry cream products sold in the local markets	June 2016– May 2017	Amol (Iran)	-1.1. -2.1. -3.2.-3.4.	-11/360 (3.1%) -No MRSA isolate was identified amongst winter samples	N/R	[82]
495 food samples from foodstuffs: 141 raw milk, 60 pasteurized milk and 80 pastry from farms and supermarkets	November 2014–November 2015	Algeria	-1.1. -2.1.1. -3.3.-3.4.	26/153 (17%): 11 raw milk, 2 pasteurized milk, and 1 pastry	SCC*mec* IV (9 raw milk, 2 pasteurized milk and 1 pastry), V (1 raw milk), and II (1 raw milk)	[83]
120 samples of cheeses belonging to “traditional agri-food products”: 12 hard cheese, 20 semihard cheese and 88 fresh cheese	October 2014–May 2016	Apulia (Italy)	-1.1. -2.1.2. -3.2.	None	N/R	[84]
81 raw milk dairy products handcrafted from a total of 40 small-scale dairies: 23 raw milk, 7 curd, 39 goat or bovine cheese, 11 butter, and 1 cream	2016	Lombardy Region (Italy)	-1.1. -2.1.2. -3.3.	3/163 of *S. aureus* isolates (1.7%) (none *mec*C positive)	N/R	[85]
276 samples from different stages along cheese production process: 36 raw milk, 36 whey, 36 curd, 36 brine, 36 drying worktops, and 96 cheeses	2010–2012	Italy	-1.1. -3.3.	None	N/R	[86]
115 *Staphylococcus* spp. isolates from and milk from healthy cows on 30 farms	November 2013–March 2014	Tunisia	-1.4.4. -2.4. -3.3.-3.4.	1/24 (4.2%) of *S. aureus* isolates (*mec*A positive)	ST97/t267/agr I/SCC*mec* V	[87]
1102 breast milk samples from pediatric patients’ mothers in a university hospital	January 2015–December 2016	Shanghai (China)	-1.1. -2.3. -3.4.-3.2.	15/71 (21.1%) of *S. aureus* isolates	-ST398 (CC398)/SCC*mec* V: t034 (2) and t6606 (1) (LA-MRSA) -ST398 (CC398)/t571: SCC*mec* IV (1) and SCC*mec* V (1) (LA-MRSA) -ST59 (CC59)/SCC*mec* IV: t172 (3), t437 (1) and t3736 (1) (CA-MRSA) -ST1 (CC1)/t127/SCC*mec* IV (1) -ST188 (CC1)/t189/SCC*mec* IV (1) -ST615 (CC72)/t324/SCC*mec* IV (1) -ST88 (CC78)/NT/SCC*mec* IV (1) -ST88 (CC78)/t15319/SCC*mec* V (1)	[88]
117 raw milk samples from local shops	January–June 2015	Morogoro (Tanzania)	-1.1. -2.1. -3.2.-3.4.	2/46 (4.4%) of *S. aureus* isolates -CN-MRSA was 4.2%	-Only one of the coagulase-negative variants of MRSA was typeable: t2603 -The other 2 *mec*A positive isolates were untypable	[89]
200 samples: 100 fresh milk and 100 locally produced soft cheese (wara)	N/R	Abeokuta (Nigeria)	-1.1. -2.4. -3.4.-3.9.	50/200 (25%): 15 wara and 35 raw milk	N/R	[90]
19 samples of dairy products: pre-packaged soft and hard cheese, yogurt, and butter	January–May 2016	Epirus (Greece)	-2 methods: ·1.1. ·1.3.10. -2.1.1. -3.3.-3.4.	None	N/R	[91]
400 milk samples of bovines from dairy farms and outlets	N/R	Jabalpur (India)	-First: 1.4.3. -Second: 1.3.6. -2.5. -3.2.-3.4.	The prevalence of PVL-positive *S. aureus* was 10.5% and all were MRSA	t657, t1839, t2526, t7286, t7684	[92]
26 samples from 21 milk collection centers from various retailers	N/R	Kampala (Uganda)	-1.3.2. -2.3.2.-2.14. -3.4.-3.3.	1/26 (3.8%) (negative for *mec*A and *mec*C)	N/R	[93]
360 samples from dairy shops, grocery stores, street vendors at different markets: 100 raw milk (caw and buffalo), 120 cheese (40 of each Kariesh cheese, white cheese, and Ras cheese), 70 yoghurt, and 70 cream	January 2016–March 2017	Alexandria, (Egypt)	-1.1. -2.1.1. -3.4.-3.2.	-31/360 (8.6%): 11 raw milk, 1 Kariesh cheese, 9 white cheese, 3 ras cheese, 2 yoghurt, and 5 cream - 34/81 (42%) of *S. aureus* isolates were resistant to cefoxitin: 12 raw milk, 1 Kariesh cheese, 9 white cheese, 3 ras cheese, 2 yoghurt, and 7 cream	N/R	[94]
350 samples of milk and dairy products from retail outlets: market milk, Domiati cheese, Kareish cheese, Ras cheese, cooking butter, yoghurt, and small-scale ice cream (50 of each)	April 2017–June 2018	Assuit province (Egypt)	-1.6. -2.1.-2.4.-2.6.	54/350 (15.4%): 9 market milk, 2 Kareish cheese, 19 Ras cheese (38%), 7 cooking butter, and 17 small-scale ice cream (34%)	N/R	[95]
150 raw milk samples from retails: 80 samples from street peddlers and 70 sample from farmers	N/R	Kafr Elsheikh governorate (Egypt)	-1.7. -2.1. -3.4.-3.2.	2/5 (40%) of *S. aureus* isolates	N/R	[96]
650 milk and dairy products from several markets, delicatessens, and open bazaars: 190 raw milk samples (154 cow milk and 36 sheep milk), 180 cheeses, 125 yoghurts, 60 butters, 40 butter cream (raw), and 55 ice creams	May 2016–August 2017	Bursa (Turkey)	-1.4.3. -2.2. -3.11.-3.4.-3.2.	-12/650 (1.9%): 1 butter, 8 cheese, 1 ice cream, and 2 milk samples -148/650 (22.8%) produced typical MRSA colonies on CHROMagar MRSA II: 77 cheeses, 25 butter, 23 butter cream, 15 milk, 7 ice cream, and 1 yogurt -According to oxacillin MICs, 95 isolates (64.2%) were confirmed as phenotype-positive MRSA -53 isolates (35.8%) were regarded as oxacillin (methicillin)-susceptible *S. aureus* -Presence of the *mec*A gene in 12 MRSA strains: 3 susceptibile to oxacillin (phenotype negative but *mec*A positive MRSA and 9 oxacillin resistant (phenotype/*mec*A positive)	N/R	[97]
10 raw and pasteurized milk from retail vendors	N/R	Dhaka (Bangladesh)	-1.1. -2.1. -3.4.-3.2.	None	N/R	[98]
305 samples were collected from four dairy plants: 74 samples of dairy products (pre-packaged soft and hard cheese, yogurt, and butter)	December 2016–May 2017	Thrace and Macedonia (Greece)	-1.1. -2.1.1. -3.4.-3.3.	None	N/R	[99]
109 samples from dairy workers and dairy products (butter)	N/R	Balaju, Kathmandu (Nepal)	-1.1. -2.4. -3.5.	4/11 (36.4%) of *S. aureus* isolates in butter	N/R	[100]
49 raw milk and 47 paneer cheese from street food vendors	September 2015–May 2016	Delhi and Bareilly (India)	-2.5. -3.2.-3.1.	-7/49 (14.3%) in raw milk -None in paneer cheese	N/R	[101]
190 raw cow milk samples and 80 traditional dairy products (24 butter, 3 cheese, 24 rayeb, and 29 l’ben) from 25 dairy farms, 25 milk tanks, 5 dairy units, and 4 local markets	April–September 2014 and 2015	Tizi Ouzou (Algeria)	-1.12.1. -2.4. -3.3.-3.4.	11/270 (4.1%): 9 raw milk and 2 acidified milk (l’ben)	ST8/t024 (all isolates)	[102]
35 *S. aureus* strains: 3 cow milk, 2 goat milk, 7 sheep milk, and 8 cheese	December 2017– February 2019	N/R	-3.4.-3.5.-3.2.	None	N/R	[103]
13 *S. aureus* isolates of pasteurized milk from 258 samples	July 2011–June 2016	China	-3.5.-3.3.-3.4.	None	N/R	[104]
180 samples of milk and milk products: 34 fresh cow milk, 14 bulk milk, 66 locally fermented milk (nono), and 66 locally pasteurized milk (kindirmo)	N/R	Nasarawa (Nigeria)	-1.2. -2.1.1. -3.2.-3.4.	9/180 (5%): bulk milk (1/14, 7.1%), nono (4/66, 6.1%), kindirmo (3/66, 4.6%), and fresh milk (1/34, 2.9%)	N/R	[105]
220 dairy cows from dairy farms mastitic cows’ milk	November 2014–May 2015	Shire, Tigray (Ethiopia)	-1.1. -2.3.1.-2.4. -3.4.-3.2.	-64/220 (29.1%) were positive for bovine mastitis -21/64 (32.8%) were found CoPS -7/21 (33.3%) of the CoPS were resistant to oxacillin (phenotypically MRSA positive). -5/7 (71.4%) of them were found to carry *mec*A genes	N/R	[106]
139 foodstuff samples including dairy products (cheese, cottage, and yogurt) and meat products (sausages and hamburgers) belonging to 18 different brands from 29 stores	September 2015–October 2016	Isfahan (Iran)	-1.12. -2.1.1. -3.4.-3.2.	9.5% of *S. aureus* isolates	N/R	[107]
200 raw milks (145 from goat and 55 from sheep)	June to September	Abeokuta (Nigeria)	-1.3.10. -2.4. -3.12.-3.4.-3.9.	37/200 (18.5%): 14 (25.5%) sheep and 23 (15.9%) goat	N/R	[108]
959 samples representing eight types of animal-based foods (pork, chicken, beef, duck, lamb, aquatic products, egg, and milk) from local markets (including 21 supermarkets and 18 wet markets)	July 2018–August 2019	Shanghai (China)	-1.6. -2.1.1. -3.4.-3.2.	None	N/R	[109]
90 raw cow milk samples from unorganized farms, milk vendors and shops	July 2018–June 2019	Aizawl, Mizoram (India)	-1.7. -2.1.-2.4. -3.2.-3.4.	2/39 (5.1%) of *S. aureus* isolates	N/R	[110]
590 raw milk samples from shopping centers: 130 bovine, 120 ovine, 120 caprine, 110 camel, and 110 buffalo	2016–2017	Iran	-1.3.1. -2.1.1. -3.1.-3.2.	28/39 (71.8%) of *S. aureus* isolates: 8 bovine, 7 ovine, 4 caprine, 1 camel, and 8 buffalo	-SCC*mec* IVa (8/28, 29.6%, CA-MRSA): 3 bovine, 1 ovine, 1 caprine, and 3 buffalo -V (7/28, 25%, CA-MRSA): 1 bovine, 2 ovine, 1 caprine, and 3 buffalo -III (4/28, 14.8%, HA-MRSA): 2 bovine, 1 ovine, and 1 buffalo -IVb (3/28, 11.1%, CA-MRSA): 1 bovine, 1 ovine, and 1 caprine -IVc (2/28, 7.4%, CA-MRSA): 1 ovine and 1 caprine -I (2/28, 7.4%, HA-MRSA): 1 bovine and 1 buffalo -IVd (1/28, 3.7%, CA-MRSA): 1 ovine -II (1/28, 3.7%, HA-MRSA): 1 camel	[111]
200 samples from local markets, dairy shops and supermarkets: 100 raw milk, 50 Talaga cheese (made from pasteurized milk) and 50 Kareish cheese (made from raw milk) samples	N/R	Beni-Suef Governorate (Egypt)	-2.1. -3.2.-3.4.	4/6 (66.7%) cefoxitin resistant MDR *S. aureus* isolates	N/R	[112]
54 raw milk and 43 dairy products (23 butter, 6 rayeb, 12 l’ben, and 2 yogurt samples) from cafeteria and creameries	2017–2018	Tizi Ouzou (Algeria)	-2.1.1. -3.3.-3.4.	5/59 (8.5%) of *S. aureus* isolates (harbored the *mec*A gene): 4 rayeb and 1 raw milk	ST5: t450 (1 raw milk) and t688 (4 rayeb)	[113]
100 samples of pasteurized camel milk from different retail markets	March–May 2017	Al-Riyadh (Saudi Arabia)	-1.1. -2.1.1. -3.2.-3.5.-3.4.	10/100 (10%)	N/R	[114]
50 fresh milk and 50 fermented milk from retailing outlets		Sokoto (Nigeria)	-1.1. -2.4.-2.6. -3.2.	6/50 (12%) fresh milk and 11/50 (22%) fermented milk	N/R	[115]
210 skimmed dairy products samples from local markets: street vendor Kareish cheese, supermarket Kareish cheese, light Feta cheese, light processed cheese, light plain yoghurt, packed and unpacked skimmed milk powder (30 of each)	May 2018–August 2019	Alexandria City (Egypt)	-1.1. -2.1.1. -3.2.-3.4.	7/210 (3.3%): street vendor Kareish cheese (2/30, 6.7%), supermarket Kareish cheese (2/30, 6.7%), light Feta cheese (1/30, 3.3%), light plain yoghurt (1/30, 3.3%), and packed skimmed milk powder (1/30, 3.3%)	N/R	[116]
50 karish cheese samples	December 2018–December 2019	Al-Qalyubia governorate (Egypt)	-1.8. -2.1.-2.3.1. -3.4.-3.2.	2/19 (10.5%) of *S. aureus* isolates	N/R	[117]
100 dairy products from different farms and local grocery shops: 50 raw milk (unpasteurized) samples (origins were from 11 horses, 20 goats, 15 camels, and 4 cows) and 50 unpacked cheese samples	August 2019–March 2020	Riyadh (Saudi Arabia)	-1.5. -2.4.-2.2.-2.4.2. -3.4.-3.13.	-51/70 (72.9%) of *S. aureus* isolates: 34 from raw milk (7 horses (70%), 12 goats (75%), 11 camels (91.7%), and 4 cows (100%)) and 17 from unpacked cheese -5/51 (9.8%) of MRSA isolates were positive for *mec*A gene (neither *mec*C nor *mec*B genes were positive): 4 raw milk (2 horses and 2 camels) and 1 unpacked cheese	Among 5 *mec*A-positive isolates: -4/5 SCC*mec* type II (80%) -the type of one isolate from cheese samples could not be detected -three isolates had two types, II + V, II + IVa, and II + IVd -one isolate had three types II + III + V	[118]
140 dairy product samples (soft cheese made from raw cows’ milk) from six dairy industry sites	N/R	Jalisco (Mexico)	-2.1.1. -3.4.-3.2.	9/63 (14%) of the *S. aureus* isolates	N/R	[119]
255 dairy product samples from farms and retail markets: 175 raw milk and 80 traditionally processed dairy products (40 yogurt and 40 cottage cheese)	December 2019–May 2020	Addis Ababa (Ethiopia)	-1.3.13. -2.4. -3.4.-3.3.	20/52 (38.5%) of *S. aureus* isolates resistant to cefoxitin (only one of these isolates (5%) was positive for *mec*A gene, and none of them were positive for the *mec*C gene)	N/R	[120]
285 samples of raw milk and unpackaged artisanal dairy products from local bazaars, retail outlets, and raw milk collection areas: 50 raw milk, 50 traditional cheeses consisting of white-pickled cheese, 50 Tulum cheese, 25 yogurt, 25 butter, 20 traditional clotted cream, 50 pastry cream, and 15 traditional Maras ice cream	January 2020–April 2021	Central Anatolia and Mediterranean Regions (Turkey)	-1.1. -2.1.1. -3.3.	5/285 (1.7%): -*mec*A gene (2): raw milk and a white pickled cheese -*mec*C gene (2): two different pastry creams -both *mec*A and *mec*C genes (1): pastry cream -None of the strains from Tulum cheeses were positive with either *mec* genes	N/R	[121]
175 samples of fermented dairy products from different shops and supermarkets: 50 plain yoghurt, 50 fruit yoghurt, 50 laban rayeb, and 25 mish cheese	N/R	Dakahlia Governorate (Egypt)	-3.2.	2/10 (20%) of selected *S. aureus* isolates	N/R	[122]
30 Karish cheese (made from raw buffalo milk) from 10 markets	N/R	Mansoura district, Dakahlia Governorate (Egypt)	-1.3. -2.1.1. -3.2.-3.4.	3/5 (60%) of *S. aureus* isolates	N/R	[123]
112 samples of ‘coalho’ cheese (artisanal rennet cheese produced with raw milk) from 56 dairy producing farms	March–December 2018	State of Ceará (Brazil)	-2.1. -3.7.-3.2.	-7/69 (10.1%) of *S. aureus* isolates -5/69 (7.3%) resistance to oxacillin	N/R	[124]
380 raw milk samples from sales centers: 120 cow, 130 sheep, and 130 goat	The spring and summer of 2021	Alborz (Iran)	-1.3.13. -2.1.1. -3.1.-3.2.-3.4.	27/42 (64.3%) of *S. aureus* isolates: 10 cow, 9 sheep, and 8 goat	N/R	[125]
109 samples of dairy cow’s milk from dairy farms	July–September 2021	Probolinggo, East Java (Indonesia)	-1.14. -2.4. -3.1.-2.6.-3.2.	-5/54 (9.3%) of *S. aureus* isolates resistant to cefoxitin and oxacillin. All in ORSAB Test -2/5 (40%) were positive for the *mec*A gene	N/R	[126]
80 milk and dairy product samples from on twelve sheep and goat farms: 18 milk samples, 28 fresh cheeses, 20 ripened cheeses, and 14 yoghurts	July–November 2021	Czech Republic	-3.2.	11/27 (40.7%) positive detected SA DNA samples by PCR: 6 fresh goat cheeses, 3 ripened sheep cheeses, and 2 ripened goat cheeses	N/R	[127]
40 food products from retail establishments and supermarkets (20 of each): raw milk and Damietta cheese	November 2021–September 2022	Alexandria (Egypt)	-First: 1.4.3. -Second: 1.11.3. -2.6. -3.2.-3.4.	3/40 (7.5%): 2 raw milk and 1 Damietta cheese	N/R	[128]
700 unpasteurized raw cow’s milk samples from 20 retail outlets	February 2019–March 2020	Mansoura, Dakahliya governorate (Egypt)	-2.4. -3.4.-3.2.	37/113 (32.7%) of PVL-positive *S. aureus*	N/R	[129]
62 samples milk and fresh soft cheese from farms	N/R	Wielkopolskie and Zachodniopomorskie Provinces (Poland)	-1.1. -2.1. -3.4.-3.2.	2/39 (5.1%) of *S. aureus* isolates	N/R	[130]
69 different raw milk samples from handmade dairy products retail stores	August 2020–May 2021	Hefei, Anhui (China)	-1.3.4. -2.7. -3.2.-3.4.	6/50 (12%) of *S. aureus* isolates	-*spa* t030 (3) -t4431 (3)	[131]
110 homemade clotted cream samples from the public bazaars: 85 cow milk, 14 water buffalo milk, and 11 cow and water buffalo milk	November 2019–December 2020	Afyonkarahisar (Turkey)	-1.5. -2.1.1. -3.2.	None	N/R	[132]
328 milk samples were collected: raw milk samples were collected from dairy farmers (n = 169) and dairy vendors (n = 139) in both the states while pasteurized milk samples were only collected from milk retail outlets/grocery shops (n = 20) in Haryana	December 2016–November 2017	Haryana and Assam (India)	-1.14. -2.17. -3.1.-3.3.	-3 MRSA isolates from raw milk from vendors in Haryana -2 MRSA isolates from pasteurized milk	SCC*mec* type V	[133]
354 samples of milk and milk products from the households and local markets: 123 cow milk, 148 cheese, and 83 yoghurts	May 2020–March 2021	West Showa Zone (Ethiopia)	-1.5. -2.4. -3.4.-3.2.	None	N/R	[134]
55 dairy products from various outlets: 25 raw yogurt and 30 ice cream	November 2021–January 2022	Baghdad (Iraq)	-3.9.-3.4.	12/28 (42.9%) of *S. aureus* isolates: 8 (47.1%) raw yogurt and 4 (36.4%) were ice cream	N/R	[135]
30 raw milk and 60 cheese samples from different foodstuffs	August–November 2021	Kafr El-Sheikh governorate (Egypt)	-2.6. -3.3.	-4/30 (13.3%) raw milk -3/60 (5%) cheese (none were positive for *mec*C)	N/R	[136]
40 raw milk samples from milk stores	N/R	Faisalabad (Pakistan)	-3.4.-3.5.-3.2.	2/73 (2.7%) of *S. aureus* isolates	N/R	[137]
100 raw milk samples from the sales points	N/R	Van (Turkey)	-2.1. -3.4.-3.2.	2/48 (4.2%) of *S. aureus* isolates	N/R	[138]
380 raw milk and traditionally produced dairy samples including cow milk (50), sheep milk (40), goat milk (50), cheese (40), cream (40), butter (40), yogurt (40), doogh (40), and kashk (40) were collected from different shopping centers	September 2021–March 2022	Urmia (Iran)	-1.3.1. -2.1.1. -3.1.-3.2.-3.4.	38/60 (63.3%) of *S. aureus* isolates: 5 cow milk, 3 sheep milk, 2 goat milk, 9 cheese, 6 cream, 6 butter, 2 yogurt, 3 doogh, and 2 kashk	N/R	[139]

N/R, Not Reported. **Meaning of the numbers used in the detection method column. 1. Pre-enrichment**: 1.1. without pre-enrichment; 1.2. pre-enrichment in buffered peptone water, BPW; 1.3. pre-enrichment in tryptone soy broth, TSB (1.3.1. TSB containing 10% NaCl and 1% sodium pyruvate; 1.3.2. TSB containing 4% NaCl, 1% mannitol, 4 mg/L cefoxitin, and 75 mg/L aztreonam; 1.3.3. TSB supplemented with 75 mg/L aztreonam and 3.5 mg/L cefataxime; 1.3.4. TSB containing 7.5% NaCl; 1.3.5. TSB supplemented with 5 mg/L oxacillin; 1.3.6. TSB supplemented with 75 mg/L aztreonam and 4 mg/L cefoxitin; TSB 1.3.7. supplemented with 75 mg/L aztreonam and 3.5 mg/L cefoxitin; 1.3.8. TSB supplemented with 50 mg/L aztreonam and 3.5 mg/L cefoxitin; 1.3.9. TSB at 2× containing 15% NaCl; 1.3.10. TSB containing 6.5% NaCl; 1.3.11. TSB-YE (yeast extract) at 2×; 1.3.12. TSB supplemented with 70 mg/mL NaCl; 1.3.13. TSB containing 10% NaCl); 1.4. Mueller–Hinton Broth, MHB (1.4.1. MHB containing 6% NaCl; 1.4.2. MHB containing 7.5% NaCl; 1.4.3. MHB containing 6.5% NaCl; 1.4.4. MHB containing 10% NaCl); 1.5. Brain Heart Infusion broth, BHI (1.5.1. BHI containing 2% NaCl); 1.6. sodium chloride broth (10%); 1.7. peptone water, PW; 1.8. nutrient broth, NB (1.8.1. NB containing 7.5% NaCl and oxacillin (6 μg/mL)); 1.9. cooked meat broth; 1.10. phosphate-buffered saline; 1.11. phenol red mannitol broth, PHMB (1.11.1. PHMB containing cefoxitin (5 mg/L) and aztreonam (75 mg/L); 1.11.2. PHMB containing oxacillin (4 mg/L); 1.11.3. PHMB containing ceftizoxime (5 μg/mL) and aztreonam (75 μg/mL)); 1.12. Giolitti–Cantoni broth, GCB (1.12.1. CGB supplemented with 3.5% potassium tellurite solution); 1.13. sodium chloride broth (7.5%); 1.14. mannitol salt broth (MSB); 1.15. sodium chloride broth (6.0%). **2. Solid media:** 2.1. Baird–Parker agar, BP (2.1.1. BP supplemented with egg yolk tellurite emulsion; 2.1.2. BP supplemented with rabbit plasma fibrinogen (BP-RPF); 2.1.3. BP supplemented with egg yolk and potassium tellurite; 2.1.4. selective media); 2.2. selective MRSA agar (BBL CHROM agar MRSA; BD) MRSA chromogenic agar; 2.3. blood agar, BA (2.3.1. BA supplemented with sheep blood; 2.3.2. 5% bovine BA); 2.4. mannitol salt agar, MSA (2.4.1. MSA supplemented with 75 mg/L aztreonam and 6 mg/L oxacillin; 2.4.2. MSA supplemented with oxacillin (4.0 mg) (MSAO)); 2.5. HiChrome MeReSa agar plates with cefoxitin and methicillin supplement (HiMedia); 2.6. oxacillin-resistance-screening-agar-base (ORSAB) supplemented with oxacillin (2 mg/L); 2.7. tryptone soy agar, TSA (2.7.1. TSA with 5% sheep blood and 0.1% esculin); 2.8. chromogenic media (MRSA-Ident-Agar); 2.9. CNA plates (Columbia colistin and nalidixic acid); 2.10. Oxacillin Salt Screen Agar^®^ (MHA with 4% NaCl and 6 µg/mL oxacillin)/oxacillin agar screen sensitivity disk agar-N supplemented; 2.11. Chrom-ID MRSA agar; 2.12. Columbia agar (CA) (2.12.1. CA supplemented with 5% sheep blood); 2.13. MRSA select medium; 2.14. Brilliance MRSA Agar (chromogenic selective agar for MRSA); 2.15. MRSAselect^®^ (BioRad, Hercules, CA, USA) agar (selective chromogenic MRSA agar); 2.16. CHROMagar *S. aureus*; 2.17. *Staphylococcus* Agar (Hi-media). **3. Identification of MRSA**: 3.1. Cefoxitin (30 μg) and oxacillin (1 μg) disk diffusion susceptibility tests; 3.2. PCR-based amplification of *mec*A gene; 3.3. PCR-based amplification of *mec*A and *mec*C genes; 3.4. antimicrobial susceptibility testing; 3.5. cefoxitin disk diffusion method; 3.6. StaphType DNA microarray assay; 3.7. oxacillin disk diffusion method; 3.8. MICs of oxacillin by broth microdilution method; 3.9. MRSA latex agglutination test (penicillin-binding protein 2a latex agglutination test); 3.10. *S. aureus spa* typing; 3.11. oxacillin E-test strips; 3.12. Mueller–Hinton agar containing cefoxitin and oxacillin; 3.13. PCR-based amplification of *mec*A, *mec*B and *mec*C genes. Baird–Parker: BP; brain heart infusion: BHI; buffered peptone water: BPW; coagulase-positive *Staphylococcus*: CPS; Giollotti-Cantoni Broth: GCB; Mannitol Salt Agar: MSA; Mannitol Salt Broth: MSB; Mueller–Hinton agar: MHA; Mueller–Hinton broth: MHB; Not typable: NT; peptone water: PW; phenol red mannitol broth: PHMB; ready-to-eat: RTE; Tryptone Soya Agar: TSA; Tryptone Soya Broth: TSB.

**Table 2:** Prevalence of MRSA in bulk-tank milk (BTM) (in the absence of clarification, the prevalence is considered based on the detection of *mec*A gene).

Samples	Period	Place	Detection Method	Prevalence	Identification	Reference
20 farms of cows: one sample of each	June 2005–August 2006	Hungary	-1.1. -2.1.1. -3.4.	None	N/R	[140]
279 *S. aureus* isolates from bulk bovine milk from 279 dairy farms	July 2001	Hokkaido (Japan)	-1.1. -2.10. -3.4.-3.2.	None	N/R	[141]
70 unpasteurized milk samples from bulk milk tanks on different dairy farms	N/R	N/R	-1.8. -3.2.-3.4.	None	N/R	[142]
1061 samples from 95 farms (89 cow farms, 2 goat farms, and 4 sheep farms): 478 BTM samples	2006–2009	Czech Republic	-First: 1.4.3. -Second: 1.3.7. -2.1.-2.15. -3.2.-3.4.	22/299 (7.4%) of *S. aureus* isolates: 18 cows and 4 goats	-SCC*mec* IV: 13 cows, 4 goats (CA-MRSA) -SCC*mec* V: 5 cows (CA-MRSA)	[143]
34 BTM samples from a veterinary university goat breeding farm	June 2006–March 2008	Brno (Czech Republic)	-First: 1.4.3. -Second: 1.3.7. -2.1.-2.6. -3.2.-3.4.-3.7.	-4/34 (11.8%)	SCC*mec* IV/t064	[144]
A single BTM sample was collected from each of 542 participating operations from dairy cows, which were collected as part of the National Animal Health Monitoring System	2007	California, Idaho, New Mexico, Texas, Washington, Indiana, Iowa, Kentucky, Michigan, Minnesota, Missouri, New York, Ohio, Pennsylvania, Vermont, Virginia, and Wisconsin (USA)	-1.1. -2.2. -3.2.	None	N/R	[145]
28 samples of fresh milk were collected from 7 milking containers per 4 dairy cattle farms from commercial farms	N/R	Rooigrond, Molelwane, Lokaleng and Mogosane (South Africa)	-1.1. -2.4. -3.4.	Resistant to methicillin: -Lokaleng 93.2% and Mogosane 81.2% (both communal farms) -Rooigrond: 7% -Molelwane: 5.7%	N/R	[146]
100 BTM samples	March–September 2009	Switzerland	-First: 1.4.3. -Second: 1.11.1. -2.14. -3.2.-3.4.	None	N/R	[22]
193 Milk filters and 184 BTM samples were collected from 78 dairy production holdings supplying the farmhouse cheese sector	April–August 2005; September 2005–February 2006; March–August 2006	Ireland	-1.1. -2.1. -3.4.-3.2.	None	N/R	[147]
180 BTM samples of 180 dairy cow farms	2009	Northern Württemberg (Germany)	-First: 1.4. -Second: 1.3.7. -2.8. -3.2.	4/180 (2.2%)	N/R	[148]
*S. aureus* LGA251 and *S. aureus* LGA254 were isolated from a BTM bovine sample from a farm	May 2007	Somerset (England)	-1.1. -3.9.-3.2.-3.4.	2/2 (100%)	ST425 (CC425): t6300 (LGA251) and t6292 (LGA254)	[149]
BTM samples from dairy cows of 60 dairy herds	February–September 2010	Brandenburg, Lower Saxony and Saxony Anhalt (Germany)	-First: 1.4.3. -Second: 1.3.7. -2.14. -3.2.-3.4.	-4/60 dairy farms (6.7%) -36 MRSA isolates from bovine milk: ·5 isolates were obtained from the MRSA-positive strains of the 60 dairy farms in this study ·31 isolates originated from the Bundesinstitut für Risikobewertung (Federal Institute for Risk Assessment): 13 isolates from a national monitoring project on BTM (ZoMo 2009), and 18 other BTM isolates submitted to the National Reference Laboratory in 2009 and 2010	-CC398: t011 (22 isolates, 61%) and t034 (14 isolates, 39%) -SCC*mec* V (33 isolates, 92%), III (2), and IVa (1)	[150]
BTM samples were collected once from all three dairy cattle herds	2008	Stuttgart (Southwest Germany)	-First: 1.4.3. -Second: 1.3.7. -2.8. -3.2.-3.4.	3/3 (100%): all 3 herds	t011/SCC*mec* V (LA-MRSA)	[151]
BTM samples from 139 farms: 703 herds of cows, 1 goat herds, and 8 sheep herds	N/R	Czech and Slovak Republic	-First: 1.4.3. -Second: 1.3.7. -2.1.-2.15.-2.6. -3.2.-3.4.	-20/326 (6.1%) of *S. aureus* isolates: in cows	-SCC*mec* IV (13) -SCC*mec* V (7)	[152]
160 milk samples (32 per HACCP level)	August 2011–January 2012	Hawassa (South Ethiopia)	-1.1. -2.4. -3.4.	Resistant to oxacillin: -Bucket at farm level: 15% -From storage containers at milk collection center: 66.7% -From transportation container: 100% -After cooling at the pasteurization plant: 83.3%	N/R	[153]
150 pooled BTM samples from 50 farms	April, May, July, August, October, and November 2009	Minnesota	-First: 1.4.3. -Second: 1.11.2. -2.13.-2.9. -3.4.-3.3.	2/150 (1.3%): the herd prevalence is 4% (2/50)	-ST5/unknown *spa* type/PFGE type close to USA100/SCC*mec* II (HA- MRSA) -ST8/*spa* type t121/PFGE type USA300/SCC*mec* IVa (CA-MRSA)	[154]
635 samples from dairy cow herds were collected in the framework of the national monitoring	January 2009– August 2010	Germany	-First: 1.4.3. -Second: 1.3.7. -2.14. -3.2.-3.4.	28/635 (4.4%): 36 isolates	-CC398: t011 (22 isolates, 61%) and t034 (14 isolates, 39%) -SCC*mec* V (33 isolates, 92%), III (2) and IVa (1)	[155]
384 samples were collected from refrigerated tanks of ewe milk	April 2009–March 2010	Spain	-1.1. -2.1.2.-2.12.1. -3.2.	None	N/R	[156]
1500 BTM samples were supplied by National Milk Laboratories Ltd., in dairy cattle	January–July 2012	United Kingdom	-1.4.3. -2.14. -3.3.	Approximately 300 potential MRSA colonies were identified and subjected to PCR testing, yielding a total of 7 *mec*A MRSA isolates from 5 farms	CC398 (ST398): -t011 (6/7): 3 SCC*mec* IVa and 3 SCC*mec* V(5C2&5)c (SCC*mec* V harbouring the czrC gene in the J1 region) -t2546/IVa (1/7)	[157]
48 samples were taken one on each of the 45 dairy farms among those having experienced MRSA isolation from BTM in the previous 3 years	October–December 2010	Ragusa (South-Eastern Sicily)	-1.5.1. -2.4.1. -3.5.-3.2.	21/48 (44%)	N/R	[158]
Bulk-tank goat’s milk of 26 farms	Monthly for 3 consecutive months	Sardinia (Italy)	-1.1. -3.2.-3.8.	None	N/R	[159]
372 milk samples: 18 bulk milk		Kaduna and Zaria (Nigeria)	-1.1. -2.1. -3.4.-3.2.	18/47 (4.8%) of coagulase-positive *S. aureus* were resistant to methicillin: 2 in bulk milk	N/R	[38]
601 *S. aureus* isolates obtained from BTM samples collected from 229 dairy sheep farms	August 2008–July 2009	Ávila, Burgos, Cáceres, León, Madrid, Palencia, Salamanca, Segovia, Valladolid and Zamora (Spain)	-1.1. -2.1. -3.3.-3.4.	-1/229 farms (0.4%) (1/601 *S. aureus* isolates) contained *mec*C -3/229 farms (1.3%) (9/601 *S. aureus* isolates) tested positive for *mec*A	-ST130/SCC*mec* XI (1 *mec*C) -ST398 (3 *mec*A): SCC*mec* V (2) and SCC*mec* IVa (1)	[160]
BTM samples from 288 dairy farms (192 organic and 100 conventional farms)	March 2009–May 2011	New York, Wisconsin, and Oregon (USA)	-Two methods: ·1.1.-2.7.1.-3.2. ·Two-step: 1.4.3. and 1.11.1.-2.15. -3.3.	1/288 (0.3%) from an organic farm in New York	ST239	[161]
Bovine BTM of 465 dairy farms in England (375) and Wales (90) and 625 dairy farms in Scotland, by National Milk Laboratories Ltd.	November 2011–October 2012	Great Britain	-1.4.3. -2.14. -3.3.-3.4.-3.9.	-10/465 dairy farms were positive for *mec*C (2.2%): none in Wales and 2.7% in England -None in Scotland for *mec*C -*mec*A was detected on one farm in England (0.3%)	-ST425 (7/10 *mec*C): t6292, t742 and t6300 -CC130/t843 (3/10 *mec*C): ST130 (2) and ST2573 (a novel yqiL single locus variant of ST130) (1) -ST398 (*mec*A)	[162]
BTM samples were collected from dairy cattle farms (1 sample per farm per year): conventional and certified (these farms are allowed to sell raw milk to consumers)	2009–2012	Germany	-First: 1.4.3. -Second: 1.3.7. -Slightly modified January 2011: ·First: 1.4.1. ·Second: 1.3.8. -2.2. -3.2.-3.4.	-Conventional farms: 14/338 (4.1%, 2009) and 14/297 (4.7%, 2010) -Certified farms: 3/30 (10%, 2010)	-CC398 (29): t011 (65.5%), t034 (31%) and t1457 (3.4%) -SCC*mec* V (75.3% of the 632 isolates), IVa (18.5%), V* (3%) and not typeable (3%)	[163]
54 BTM samples from 10 farms: 23 of ovine and 31 of goat	2013–2014	Czech Republic	-1.2. -2.1.1. -3.2.	None	N/R	[164]
428 raw milk samples from bovine, ovine, caprine and bubaline farms	2011–2013	Central Italy	-1.1. -3.2.-3.4.	1/428 (0.2%): from an ovine BTM sample	ST1 (CC1, human CA- MRSA)/*spa* type t127/SCC*mec* type IVa	[46]
197 samples from 197 dairy goat farms	July–October 2012; and summer 2013	Lombardy Region (Northern Italy)	-Two methods: ·1.1.-2.3.1.-2.1.2.-3.7.-3.3. ·Two-step: 1.4.2. and 1.3.5.-2.14.-3.3.	-4/197 (2%): 3 detected by direct plating, and 1 detected following enrichment -Persistence of MRSA on the 4 MRSA-positive farms was investigated 1 year after the first examination: 3 BTM samples retested positive for *S. aureus*, but only 1 was still contaminated by MRSA	-ST398/SCC*mec* V/t899 (3) (LA-MRSA) -ST1 (CC1)/SCC*mec* IVa/t127 (1) (LA-MRSA)	[165]
282 raw milk samples collected from BTM	April–October 2012	Milan and Monza-Brianza (Italy)	-1.1. -2.1.2. -3.4.	5/282 (1.7%)	- SCC*mec* IV (1) and V (2) (CA-MRSA) -SCC*mec* I and II (2) (HA-MRSA)	[53]
223 raw BTM samples from 39 goat farms	October 2011–September 2013	Poland	-1.1. -2.1.2. -3.4.-3.2.	None	N/R	[166]
248 raw milk samples from bulk tanks at 12 dairy farms	July 2010–October 2012	Shanghai (China)	-1.1. -3.2.	7/58 (12.1%) of *S. aureus* isolates	-ST965 (CC5)/t062/*agr* II (3) (HA-MRSA) -ST9 (CC9)/t899/*agr* II (2) (LA-MRSA) -ST59 (CC59)/*agr* I (2) (CA-MRSA)	[167]
261 BTM samples collected during official monitoring activities	2008	Italy	-1.1. -3.5.-3.4.	2/261 (0.8%)	ST398/*spa*-type t899: SCC*mec* type IV and V	[56]
A BTM sample at the end of the milking procedures from dairy sheep farm previously identified as MRSA positive by testing BTM	May 2014	Rome (Italy)	-Two methods: ·1.1.-2.1.2.-2.2. ·1.4.3.-2.2. -3.3.-3.4.	1/1 (100%): tested positive, after both enrichment and by direct plating	ST1 (CC1, human CA- MRSA)/*spa* type t127/SCC*mec* type IVa	[168]
162 BTM samples from sheep and/or goat farms: 96 sheep milk, 49 goat milk, and 17 mixed sheep and goat milk	January–July 2014	Apulia and Basilicata (Southern Italy)	-1.4.3. -2.15.-2.10. -3.1.-3.11.-3.2.-3.4.	2/162 (1.2%): 1 from a sheep farm of Apulia and 1 from a goat farm of Basilicata	- ST1/t127/SCC*mec* IVa (sheep) - ST398/t1255/SCC*mec* V (LA-MRSA) (goat)	[169]
844 BTM samples from dairy cattle herds	July 2012– October 2013	Lombardy Region (Northern Italy)	-Two methods: ·1.1.-2.1.2.-3.5.-3.3. ·Two-step: 1.4.2. and 1.3.5.-2.14.-3.3.	-32/844 (3.8%) -15 strains were isolated by direct plating, 11 were detected following enrichment and 6 by both methods	-ST398 (CC398) (14) (LA-MRSA)/SCC*mec* V: t899 (7), t034 (2), t011 (2) and t108 (1); and SCC*mec* IVb/t899 (2) -ST97 (CC97) (7)/SCC*mec* V: t1730 (3), t4795 (2), t2421 (1) and t9295 (1) -ST1(CC1)/IVa/t127 (7) (LA-MRSA) -ST5 (CC5)/V: t535 (1) and t548 (1) -ST461 (CC5)/V/t688 (1) -ST3211 (CC22)/IV/t309 (1)	[170]
BTM samples from 224 dairy cows farms (that resulted positive for *S. aureus* at the previous annual screening)	February–March 2011	Brescia, Bergamo, and Mantova (Northern Italy)	-First: 1.4.3. -Second: 1.3.7. -2.2. -3.3.-3.4.	9/224 (4%)	-ST398: t899 (3), t001 (1), and t108 (1) -ST97 (CC97): t4795 (3) and t9305 (1)	[171]
486 BTM samples of cow’s milk	September 2012–April 2013	Apulia and Basilicata (southern Italy)	-1.4.3. -2.15. -3.2.-3.1.-2.10.-3.11.-3.4.	12/486 (2.5%)	-ST1/SCC*mec* IVa (3 isolates; 25%): t127 (2) (CA-MRSA) and t174 (1) -ST8/unknown *spa* types: SCC*mec* IVa and V (2, 16.6%) -ST398/IVa/t899 (1) - ST398/V/t011 (1) -ST5/V/t688 (1) -ST45/IVa/t015 (1) -ST71/V/t524 (1) -ST88/IVa/t786 (1) -ST2781/V/t1730 (1)	[172]
140 ovine and 35 caprine BTM samples from farms	February–May 2014	Thessaly (Greece)	-1.1. -2.1.1. -3.4.-3.2.	1 isolate in an ovine milk sample	t4038	[173]
165 samples: 32 samples of raw milk, 38 swabs of udder skin, 38 samples of milk from cows with subclinical mastitis, and 57 environmental samples (10 swabs of milking machines, 13 swabs of milk tanks, 10 samples of animal feed, 24 swabs from floors of farm buildings) were collected from 5 dairy farms	2014–2016	Sumy (Ukraine)	-1.1. -2.1.1. -3.4.-3.2.	10/62 of *S. aureus* isolates (16.1%)	N/R	[174]
50 raw milk samples: 20 cows’, 15 goats’, and 15 ewes’ bulk milk	August 2014–May 2015	Hatay (Turkey)	-Homogenized: 1.7. -1.1. -2.1.1. -3.4.-3.2.	None	N/R	[71]
50 cow BTM samples	N/R	Balikesir (Turkey)	-1.4.3. -2.2. -3.9.-3.2.-3.4.	2/50 (4%) by Slidex MRSA latex agglutination test	N/R	[74]
286 BTM samples from 286 dairy sheep farms	January–May 2012	Lazio (Italy)	-1.1. -2.1.2. -3.5.-3.3.-3.4.	2/286 (0.7%): in 2 samples from 2 different farms (1 *mec*A and 1 *mec*C)	-ST1 (CC1)/SCC*mec* IVa/ t127 (*mec*A positive) -ST130 (CC130)/SCC*mec* XI/t843 (*mec*C positive)	[175]
51 *S. aureus* strains were collected from 51 samples of six dairy sheep farms, including two farms with artisan dairy facilities, with a history of MRSA in BTM: 11 BTM samples	2013–2015	Latium (Italy)	-1.1. -2.1.2. -3.3.	27/51 (52.9%): 10 BTM	t127 (7), t1166 (1), t1773 (1) and t044 (1)	[78]
57 bovine BTM samples from 12 bovine dairy farms	September–October 2017	Jenin district (West Bank-Palestine)	-1.3. -2.4. -3.4.-3.2.	-24/39 (61.5%) of *S. aureus* isolates -7/12 (58.3%) of farms had MRSA contaminated BTM samples -66.7% of *S. aureus* isolates were identified as MRSA by the cefoxitin disk diffusion method -45.6% of bovine BTM samples were contaminated with MRSA	N/R	[176]
208 BTM samples from 117 dairy farms: 44 cattle, 47 sheep, and 26 goat	December 2015–March 2016	Jordan	-1.4.3. -2.14. -3.3.-3.4.	-54/208 (26%): 31.8% of cattle, 29.8% of sheep, and 11.5% of goat dairy farms (86 isolates) (none positive for *mec*C) -Prevalence bunk tank level: cattle 20% (80), sheep 38.4% (87), and goats 11.9% (42). Total 26% (209). -Prevalence farm-level: cattle 31.8% (44), sheep 29.8% (47), and goats 11.5% (26). Total 26.5% (117).	N/R	[177]
165 fresh bulk milk samples from local bovine herds	N/R	Ibarapa, Oyo and Oke-Ogun (Nigeria)	N/R	7.9% isolates identified as MSRA	N/R	[178]
36 BTM samples: 10 bovine, 19 ovine, 5 caprine, and 2 mixed ovine and caprine	January–May 2016	Epirus (Greece)	-Two methods: ·1.1. ·1.3.10. -2.1.1. -3.3.-3.4.	1/10 (10%) bovine BTM samples	*spa* type t127	[91]
Samples were collected from 675 dairy herds (372 conventional and 303 organic)	2014	Germany	·First: 1.4.1. ·Second: 1.3.8. -2.2. -3.3.-3.4.	-36/372 (9.7%) conventional -5/303 (1.7%) organic	-Most isolates (38/41) CC398 (LA-MRSA) -Conventional (36): ·CC398: t011 (19), t034 (10), t108 (1), t2346 (1), t2576 (1), t4677 (1) and NT (1) ·CC1/t127 (1) ·CC9/t1430 (1) -Organic (5): ·CC398: t011 (2) and t034 (2) ·CC22/t790 (1)	[179]
286 BTM samples from 286 dairy farms	2014–2015	Denmark	-1.4.3. -2.14. -3.8.-3.3.	8/286 (2.8%)	-CC398/SCC*mec* Vc/t034/*mec*A positive (LA-MRSA, 7/8) -CC130/t843/*mec*C (LA-MRSA, 1/8)	[180]
18 raw BTM samples from four dairy plants: 9 bovine, 7 ovine, and 2 caprine	December 2016–May 2017	Thrace and Macedonia (Greece)	-1.1. -2.1.1. -3.4.-3.3.	2/18 (11.1%): 1 ovine raw milk (1/7, 14.3%) and 1 bovine raw milk (1/9, 11.1%)	-t044 (ovine raw milk) -t337 (bovine raw milk)	[99]
150 BTM samples from healthy cows from 63 different dairy farms	N/R	Shanghai (China)	-1.13. -2.16.-2.1. -3.2.-3.4.	2/79 (2.5%) of *S. aureus* isolates	LA-MRSA ST398/SCC*mec* type V/*spa* type t034 (2)	[181]
363 bovine BTM samples from 363 dairy farms	September 2015–February 2016	England and Wales	-1.4.3. -2.14. -3.5.-3.3.	3/363 (0.8%)	-SCC*mec* IVa(2B) (1 *mec*A): ST398 - SCC*mec* XI(8E) (2 *mec*C): ST130 and ST425	[182]
123 raw milk samples (99 from goats and 24 from ewes) collected on farm level from tank milk or from milk churn	February–May 2019	Switzerland	-First: 1.4.3. -Second: 1.3.3. -2.14. -3.6.-3.2.	4 goats’ milk samples (4%), but from none of the ewes’ milk samples	- CC398 (3, *mec*A) - CC8 (1, *mec*A)	[183]
75 buffalo BTM samples from 75 farms	April 2017–May 2018	Italy	-1.4.3. -2.15. -3.2.-3.1.-2.10.-3.11.-3.4.	3/75 (4%)	-ST1/t127/Va (2) -ST72/t3092/V (1) (CA-MRSA)	[184]
50 bovine BTM samples from dairy farms	2017–2018	Fayoum city (Egypt)	-1.7. -2.1. -3.4.-3.7.-3.2.	-4/50 (8%) -9/50 (18%) resistant to oxacillin	N/R	[185]
697 BTM samples from dairy farms	January 2017–April 2018	England and Wales (Great Britain)	-1.15. -2.14. -3.3.-3.4.	6/697 (0.9%): 4 *mec*C and 2 *mec*A	4 *mec*C: -ST425 (3): t6292 (2) and t10855 (1) - ST4652/t843 (1) 2 *mec*A: - ST398/t034 - ST5/t002	[186]
365 pooled BTM samples (189 winter and 176 summer samples) from 189 herds	January 2016–January 2017	Wisconsin, Minnesota, California, Ohio, Indiana, New York, South Dakota, Iowa, Michigan, Idaho, Maine, Montana, Texas, Georgia, Vermont, Florida and Washington (United States)	-1.1. -2.3. -3.2.	1/124 (0.8%) of *S. aureus* isolates (0.5%; 1 of 189 herds)	ST72-CC8, *spa* type t126	[187]
418 pooled BTM samples from 418 different dairy farms	September 2015–July 2017	Shandong (China)	-1.6. -2.1.3.-2.3.1. -3.4.-3.3.	3/418 (0.7%) cefoxitin-resistant isolates (only one isolate carried *mec*A, and none of the isolates carried *mec*C)	-ST97, ST2779, and ST3191 -t002, t267, and t437	[13]
290 samples of raw cow’s milk from milk storage tanks in cattle farms and milk supply places	May–December 2016	Urmia city (Iran)	-3.7.-3.2.	-7/44 (16%) of *S. aureus* isolates were resistant to oxacillin: 1 from the tanks and 6 from the raw milk supply centers -5 isolates had *mec*A gene: 4 milk supply centers and 1 milk tanks	N/R	[188]
97 bulk tank sheep’s milk samples from a different farm	January–February 2020	Tuscany and Lazio (Italy)	-1.4.3. -2.4.2. -3.4.-3.3.	-1/97 (1%) -None of the isolates were positive for *mec*C gene	*spa*-type t127	[189]

For interpretation, see Table 1.

## 3. Discussion

The prevalence of MRSA in milk and dairy products varies greatly between studies, which could reflect the heterogeneity of both the methods used and the type of samples analyzed (geographic origin, size, manufacturing technology, use of raw or pasteurized milk, sample storage, and handling) [12]. 

The source of *S. aureus* contamination of raw milk on dairy farms could be from the animals themselves or from the product processing environment, such as animal housing or water. Greater contamination is expected the lower the personal hygiene and that of utensils [190].

*S. aureus* is one of the main causative agents of subclinical mastitis worldwide and is also frequently isolated from animals with clinical mastitis [191]. Most MRSA infections on dairy farms are related to the continued use of antibiotics on animals and physical contact between dairy cows and milkers. Therefore, it is possible that this infection is transmitted through farmers’ hands during milking [192].

Bulk tank milk (BTM) analysis is a simple, rapid, and economical alternative or complement to determine the microbiological quality of milk quarter samples. Quantitative BTM testing is useful for pathogens whose increased presence in bulk milk can lead to a higher risk of the bacteria spreading and a higher risk of new infections on the farm. BTM tests can mainly be used for the early detection of infection risks from microorganisms that are transmitted during milking and which, due to their high rate of spread, represent a massive threat to udder health on dairy farms. In the case of *S. aureus*, the risk of infected cows increases with increasing herd size and the associated increase in milkings per group. Thus, a limit higher than 10 cfu of *S. aureus*/mL of bulk milk is considered to considerably increase the risk of infection [193]. In most of the articles reviewed, MRSA is confirmed by identification of the *mec*A gene by PCR, and in some articles the *mec*C gene is included, allowing greater recovery of positive samples. The PCR method cannot be performed in all laboratories due to cost and resource limitations. To find MRSA, the limitations of PCR can be circumvented by using the disk diffusion method with cefoxitin and oxacillin, followed by investigation using ORSAB [192]. 

In a large number of articles, the typification of MRSA strains is not performed, especially in the case of milk in bulk tanker samples. CA-MRSA strains carry SCC*mec* types IV and V but HA-MRSA strains carry SCC*mec* types I, II, and III, and SCC*mec* V and SCC*mec* IV are the most common types between them. LA-MRSA clones are various between countries, e.g., the CC9 clone was uncovered in Asia but the CC398 clone was uncovered in Europe and the USA [118]. The *mec*C gene located in the new SCC*mec* type XI has been observed. LA-MRSA strains evolved independently from common HA-MRSA or CA-MRSA usually found in humans and mainly belong to ST398, as the predominant MRSA type in dairy herds in Europe. MRSA isolates from intramammary infections of ruminants are also associated with ST1, ST97, ST5, and ST130 [14]. 

## 4. Materials and Methods

Various databases, including Web of Science, Scopus, Pubmed, and ScienceDirect, were consulted so as to produce an inventory of all relevant studies on the prevalence of MRSA in milk and dairy products. All work carried out between January 2001 and February 2024 was reviewed. The key words used to search for articles were “prevalence or incidence”, “MRSA”, “methicillin-resistant *Staphylococcus aureus*”, followed by the terms for each of the food groups evaluated. No language, article type, or text availability conditions were applied. A total of 125 items were identified for dairy products and 61 for bulk-tank milk (BTM).

The results were ordered by year of publication and, within each year, by alphabetical order of the authors of the articles. The dates and places of the studies, the prevalence of MRSA (in the absence of any additional clarification, only determination of the *mec*A gene was considered in the detection of the microorganism), and the typing of the MRSA strains found in the study were analyzed. A numerical code, explained at the foot of Table 1 and Table 2, was created to pinpoint which protocol among the different techniques for identifying MRSA had been followed in each of the pieces of research described.

## 5. Conclusions

In the majority of investigations carried out into milk and dairy products, MRSA was detected, although with a low prevalence, generally less than 5%. In order to avoid strain identification errors, the *mec*B and *mec*C genes should be determined as well as the *mec*A gene. The great diversity of methods used for the determination of MRSA makes comparisons between studies difficult. This points to a need for a standardized protocol for the study of this microorganism in foods.
